# Percutaneous extracapsular repair as a cost-effective alternative for treating cranial cruciate ligament deficiency in dogs

**DOI:** 10.3389/fvets.2025.1616484

**Published:** 2025-07-31

**Authors:** Dominique J. Griffon, Ayman Mostafa, Etienne Griffon, David J. Schaeffer

**Affiliations:** ^1^Atlantic College of Veterinary Medicine, Prince Edward Island University, Charlottetown, PEI, Canada; ^2^College of Veterinary Medicine, Western University of Health Sciences, Pomona, CA, United States; ^3^Department of Small Animal Surgery and Radiology, College of Veterinary Medicine, Cairo University, Giza, Egypt; ^4^D'Amore McKim School of Business, Northeastern University, Boston, MA, United States; ^5^College of Veterinary Medicine, University of Illinois Urbana-Champaign, Champaign, IL, United States

**Keywords:** access to veterinary care, stifle, cranial cruciate ligament, gait analysis, extracapsular repair

## Abstract

**Objective:**

To evaluate the duration of surgery, cost, and outcomes associated with percutaneous placement of a lateral fabellotibial suture (pLFS) in dogs with unilateral cranial cruciate ligament deficiency (CCLD) treated in a low-cost clinical setting.

**Study design:**

Block randomized prospective clinical trial on 24 dogs.

**Methods:**

Dogs underwent an exploratory arthrotomy and extracapsular repair (ECR) or pLFS. Intraoperative findings, cost, and duration of surgery were recorded. Each dog was evaluated preoperatively and 2, 6, and 12 weeks after surgery with a standardized owners’ questionnaire, clinical examination, radiography, thermal imaging, and pressure gait analysis. Outcomes were evaluated longitudinally within dogs and between groups.

**Results:**

The pLFS technique was faster (about 20 min) and more cost-effective (by about 100$) than the ECR repair (*p* = 0.01 and 0.03). The only major complication consisted of a surgical infection requiring revision surgery after pLFS. Limbs had less reduction in thigh diameter 2 weeks after pLFS (−1.2 ± 0.05%) compared to those treated with ECR (−5.5 ± 0.04%, *p* = 0.03). The pressure placed on the operated limb averaged 78% of that of the contralateral limb 2 weeks after pLFS compared to 43% after ECR (*p* = 0.04). Similar results were obtained when comparing the ratio of activated sensors and relative stance time. No differences in owner assessment, radiographic progression of osteoarthritis, lameness scores, girth diameter, and gait analysis at the walk and trot were detected between groups.

**Conclusion:**

The pLFS was faster, more cost-effective, and improved limb function at 2 weeks compared to the ECR. These results justify the consideration of the pLFS as a low-cost alternative to ECR.

## Introduction

Cranial cruciate ligament deficiency (CCLD) is the leading cause of lameness and degenerative joint disease (DJD) in the canine stifle (1). In the United States, CCLD generates the highest financial burden of all small animal orthopedic conditions (2, 3) due to the prevalence of this condition and the increasing cost of its treatment (4). Tibial osteotomies have gained popularity but are more expensive than traditional extracapsular repairs (ECRs), first described in 1970 (5). The cost and invasiveness of surgery remain barriers preventing their application in all affected dogs. These limitations are especially relevant in socioeconomically disadvantaged populations and shelters, where excessive costs may lead to euthanasia or complicate adoption (6). One of the main barriers to accessing veterinary care consists of its associated costs (7). Trends toward specialization and corporate-ownership of veterinary medicine have generated concerns over access to veterinary care, justifying a recent emphasis on the “spectrum of care,” also termed “contextualized care” (8). This concept can be defined as “providing a continuum of acceptable care that considers available evidence-based medicine while remaining responsive to client expectations and financial limitations” (9).

The traditional ECR includes an exploratory arthrotomy to excise the remnants of the CCL and treat any meniscal disease. The technique relies on a figure-of-eight suture placed around the lateral fabella, under the patellar tendon, and through a tunnel in the proximal tibial tuberosity to address the cranial drawer and internal rotational instability induced by CCLD (5). The lateral joint capsule and Fascia Lata are imbricated before the routine skin closure. This technique does not require specialized equipment and is technically less challenging than tibial osteotomies (10). Complications associated with ECR also seem fewer and more minor than those associated with tibial osteotomies (11). However, ECR may still be too costly, and dogs generally do not start using the operated limb for several weeks after ECR, which could delay adoption and generate additional housing costs for shelters and foster homes. This study aimed to validate a simple surgical treatment for canine CCLD that would be cost-effective while allowing an earlier return to limb function.

The percutaneous ECR (pLFS) is similar to the traditional ECR except that the suture is passed percutaneously through two small incisions, and the stifle joint capsule is not incised. The technical ease and biomechanical properties of the proposed pLFS repair have been reported (12, 13). In the clinical setting, the authors observed that dogs treated with pLFS were generally toe-touching within 48 h and seemed to start weight-bearing faster than those treated with arthrotomy and traditional ECR. This earlier return to function would be advantageous in shelters and low-cost clinics. However, these outcomes have not been measured with standardized and objective protocols.

This study aimed to evaluate the outcomes of dogs treated for CCLD with a pLFS in a study intending to treat in a low-cost clinical setting. We hypothesized that the pLFS repair would be faster, reduce postoperative inflammation evaluated with thermal imaging measurements, and improve limb function 2 weeks after surgery compared to the traditional ECR.

## Methods

### Population

Dogs with CCLD were recruited from the Pomona, CA community between April 2021 and June 2023. According to the AVMA and U. S. Census Bureau, Pomona residents earned an average of $53,281/year/household during this period, with 20.7% of households below the federal poverty level. The IACUC of Western University of Health Sciences approved all procedures. The study was advertised on the College website, via emails to the campus community, local postings of flyers, and letters to veterinarians within a 50-mile radius. Owners signed an informed consent form and left a $200 deposit, which was returned at the last recheck. All diagnostics, treatments, and rechecks related to the CCLD (including complications) were performed at no cost to the owners.

Dogs were included if they were healthy adults weighing less than 60 Kg, with unilateral lameness of a hindlimb due to CCLD and no known history of trauma (14). Deficiency of the cranial cruciate ligament was diagnosed based on clinical evidence of cranial drawer and radiographic evidence of effusion in the affected stifle, with no evidence of other stifle disease. Meniscal disease was not used as a criterion for inclusion or exclusion because this condition is commonly secondary to CCLD and would be expected to develop randomly in dogs in each study group (10). Dogs with concurrent patellar luxation, symmetrical lameness, or neurological abnormalities were excluded. Dogs classified as status 3 or above, according to the American Society of Anesthesiologists (15), were excluded from the study. Dogs with significant complications, defined as requiring revision surgery, were treated for their complications; only the data collected prior to the complication were included in the statistical analyses. All data collected on dogs with minor complications, defined as resolved spontaneously or with medical treatment, were included in the statistical analysis.

### Perioperative management

The anesthesia protocols used in this study follow the 2020 AAHA anesthesia and monitoring guidelines (15). Routine blood work was evaluated in each dog before anesthesia. Dogs undergoing ECR were premedicated with dexmedetomidine (0.005 mg/kg) and butorphanol (0.2 mg/kg IM) or morphine (0.5 mg/kg IM). Anesthesia was induced with propofol (4 mg/kg IV) and maintained with sevoflurane. The anesthesia for dogs undergoing pLFS was designed to meet the needs for a shorter and less invasive procedure, as well as feasibility in low-cost settings: sedation and induction relied on dexmedetomidine (0.012 mg/kg), butorphanol (0.24 mg/kg) and ketamine (2.4 mg/kg). Sevoflurane was administered if signs consistent with a light plane (plane 1) of anesthesia were observed. All dogs received carprofen (4.4 mg/kg SC) after induction for preemptive analgesia, cephazolin (22 mg/kg IV) for antimicrobial prophylaxis (repeated every 90 min if indicated), and intraoperative IV fluids. A modified Robert Jones bandage was maintained for 24 h after surgery. Postoperative analgesia consisted of buprenorphine (0.02 mg/kg SC) after recovery. Dogs were discharged the day after surgery with 5 days of tramadol (4 mg/kg, TID, PO) and 10 days of carprofen (2.2. mg/kg BID, PO). All owners received the same instructions for exercise restriction and home rehabilitation.

### Surgical treatments

Following block randomization, dogs were assigned to the ECR or pLFS through drawing. All surgeries were performed by the same DACVS (DG), assisted by a veterinary student. The veterinary student was supervised while drilling the tibial tunnel and closing the subcutaneous and cutaneous layers. The same veterinary technician (ZM) monitored the anesthesia of dogs in the study.

**Figure 1:**
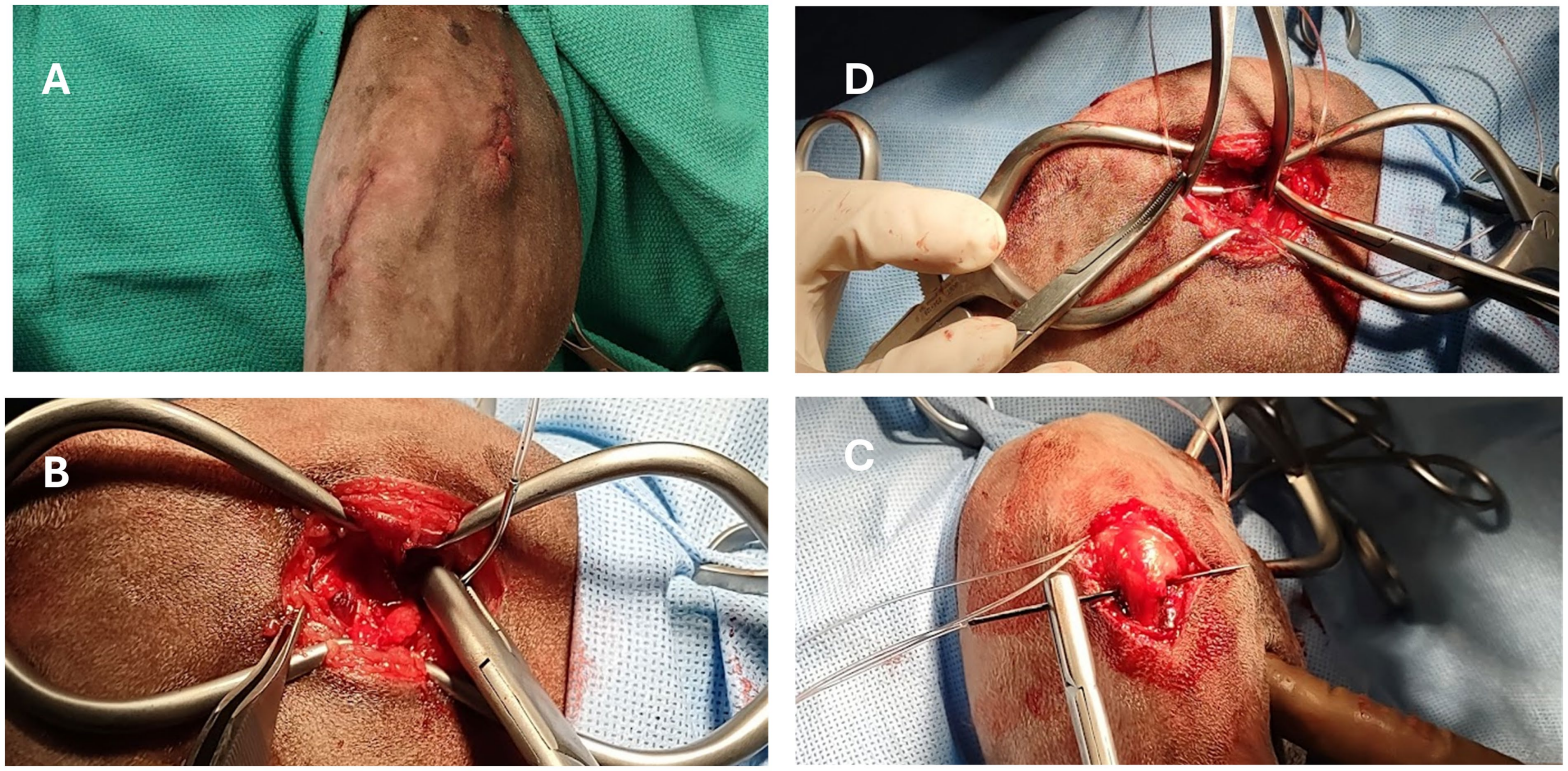
Intraoperative images of a dog undergoing percutaneous placement of a lateral fabellar suture (pLFS). **(A)** Two small skin incisions are created over the lateral fabella and proximomedial tibial tuberosity. **(B)** Placement of a loop of nylon suture around the fabella. **(C)** The suture is tunneled under the Fascia Lata and patellar tendon in a lateromedial direction before passing through the tibial tunnel. **(D)** The loop is cut to create two strands, each tightened and crimped under tension.

The pLFS was performed as described by Biskup and Griffon (12, 13), (Figure 1). Two small incisions were created over the lateral fabella and the proximal tibia. A pin of appropriate size was placed in a Jacob’s chuck to create a tunnel in the proximal tibia about 5-7 mm distal to the joint and caudal to the cranial aspect of the tibial tuberosity. The proximal incision was extended into the Fascia Lata, to expose the lateral fabella. A loop of individually swaged-on double nylon leader line (NLL, Securos Inc., Fiskdale, MA) was placed through the fibrous origin of the lateral head of the gastrocnemius muscle, slightly proximal to the fabella (16) and exiting distal to the fabella, close to the femur. The weight of the suture approximated the dog’s weight rounded up to the closest size available. Secure suture placement was confirmed before passing the suture under the Fascia Lata and subcutaneous tissues to exit through the distal incision. The suture was passed under the patellar tendon and through the tibial tunnel. The suture was passed under the soft tissues to exit into the proximal incision. Strands of NLL 18 kg and larger were tied with a crimp clamp system of equivalent size (Securos Inc., Fiskdale, MA). The NLL was tensioned with a universal tensioning device (Securos Inc., Fiskdale, MA) until the cranial drawer was eliminated while maintaining the stifle in slight flexion (≥90° angle). The Fascia Lata, subcutaneous tissue, and skin were closed with appositional continuous patterns of absorbable monofilament sutures.

The control group underwent ECR (14), starting with a craniomedial skin incision over the stifle. Subcutaneous tissues were dissected to allow craniolateral exploratory arthrotomy of the affected stifle. Remnants of the CCL were excised before evaluating the menisci. Meniscal tears were treated by partial meniscectomy. Before exposing the lateral fabella, the joint capsule was closed with a monofilament in an imbricating interrupted pattern. The same type of nylon suture was placed as described for the pLFS. The suture was placed in a figure-of-eight pattern, according to the same landmarks as the pLFS, the only difference consisting of the open surgical approach. The Fascia Lata was imbricated with an interrupted pattern before routine closure of the subcutaneous and intradermal tissues with appositional continuous patterns of absorbable monofilament sutures.

The duration of each surgery was recorded, and the cost was calculated based on consumables (without markup), a professional fee of 170$/hour, and a set fee for technical assistance.

### Measures of outcome

#### Owners’ assessments

The same owner completed the standardized Canine Brief Pain Inventory (10) preoperatively and 2, 6, and 12 weeks after surgery. Scores increased from 0 to 100 with loss of function, pain, and impact on the quality of life.

#### Clinical examinations

Signalment and history were obtained before preoperative physical examination, including neurological and orthopedic assessments performed by the same investigator throughout the study (DG). The lameness was scored from 0 (normal gait) to 5 (continuously non-weight-bearing). The cranial drawer was measured (mm) along with goniometric measurements (°) of the flexion and extension of the stifle. The length of the femur was measured from the greater trochanter to the lateral fabella. Girth circumference (cm) was measured on both pelvic limbs throughout the study at 70% of this distance for each thigh (17, 18). The distance from the fibular head to the lateral malleolus was measured. Girth circumference (cm) was measured throughout the study at 25% of this distance for each crus. The same investigator (ZM) performed goniometric and girth measurements throughout the study. Clinical examinations were repeated at 24 h, 2, 6, and 12 weeks after surgery. Any complications were noted and treated.

#### Radiographic examinations

Radiographs of the stifle were obtained before and repeated immediately, 6, and 12 weeks after surgery, under sedation or anesthesia (postoperative radiographs). Lateral and craniocaudal views of the stifles were assessed for abnormalities and implant placement. Radiographic studies obtained at each time point were anonymized and randomized to ensure masked scoring for osteoarthritis (OA) by the same investigator (AM) throughout the study. The scoring system (19) includes 32 radiographic features of OA, each scored from 0 (absent) to 3 (severe).

#### Thermal imaging

**Figure 2:**
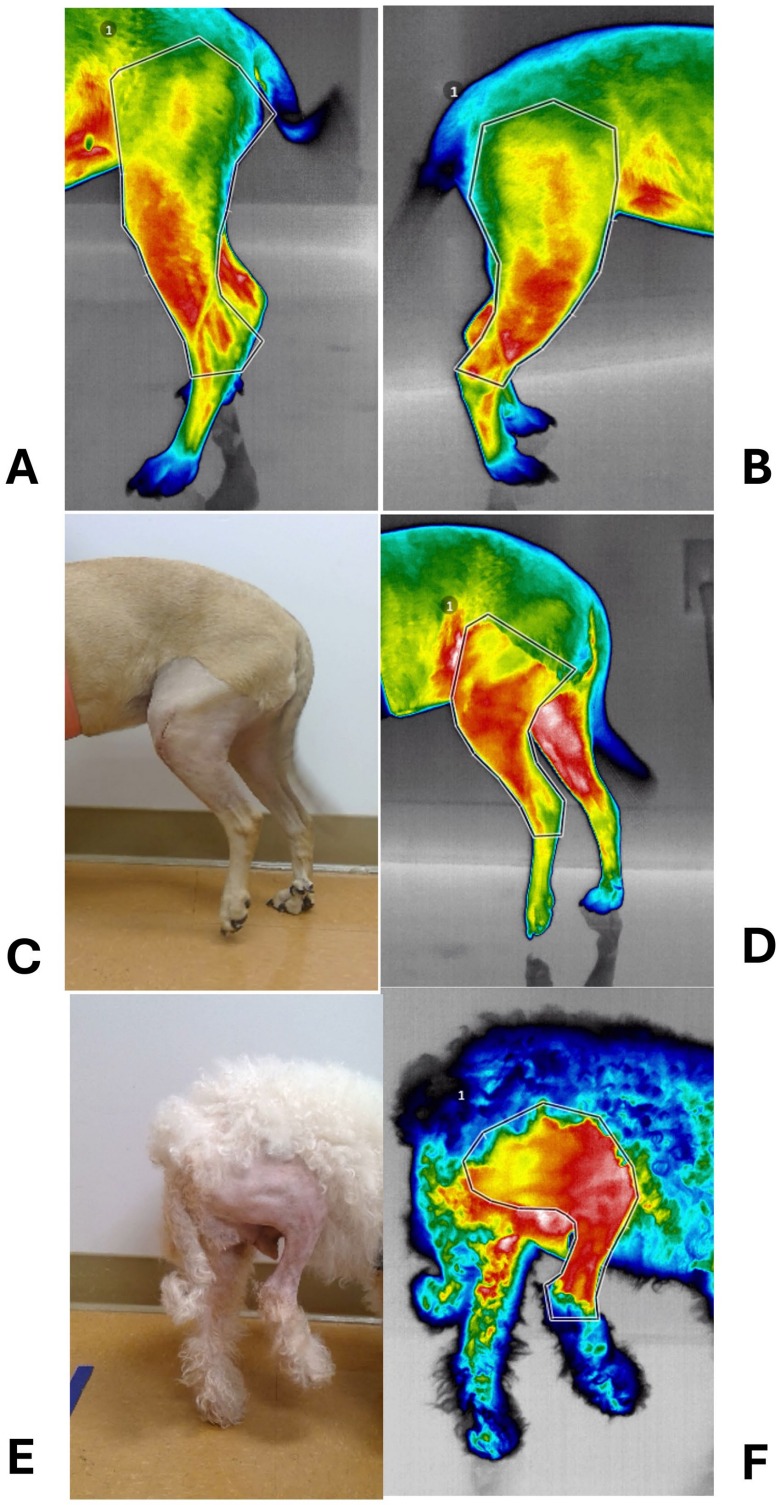
Photographs of thermal imaging studies. **(A)** Preoperative thermal image of a dog with left cranial cruciate ligament deficiency (CCLD). **(B)** Thermal image of the contralateral, normal limb. **(C)** Photograph of the left limb shown in A, 24 h after percutaneous placement of a lateral fabellotibial suture. **(D)** Corresponding thermal image. **(E)** Photograph of a right limb 24 h after traditional extracapsular repair of CCL. **(F)** Corresponding thermal image.

Thermal imaging (640 Digatherm Thermal Imaging System, Digatherm LLC, Beaumont, TX) was performed on standing dogs by the same investigator (ZM) and in the same room, before, 1 day, and 2, 6, and 12 weeks after surgery. Two images of the lateral aspect of each limb obtained at 2 and 4 feet from the dog were cropped to extend from mid-thigh to mid-crus (Figure 2). Dogs were not clipped for preoperative imaging or at any time during the study. For statistical comparisons, the maximum temperatures measured on each stifle were averaged (°C) and normalized to the average temperature of the corresponding area over the contralateral stifle.

#### Pressure gait analysis

Limb function was assessed at a walk on an instrumented pressure mat (GAIT4Dog®, CIR Systems, Franklin, NJ) before, at 2, 6, and 12 weeks after surgery, and at the trot at 12 weeks postoperatively. The same investigator (ZM) collected the data. A minimum of three valid recordings were obtained at each time. A recording was considered valid if the gait seemed consistent (no skipping or hopping), the movement forward seemed consistent (no sudden stop or acceleration), all four paws made contact with the central portion of the mat, and the head faced forward at all times. Velocity (controlled/s), cadence (steps/min), stance time (s), total pressure index (TPI, %), and number of activated sensors were measured. Velocity and cadence were not standardized between dogs to accommodate size and body conformation variations. Instead, a trial at the walk was considered valid if the velocity and cadence varied within 10% of previous recordings obtained on the same dog throughout the study. Three trials at the trot were considered valid if their velocity and cadence were within 10% of each other at 12 weeks. Symmetry indices for the TPI, stance time, and number of activated sensors were calculated as ratios between the operated and contralateral limbs (20).

### Data analysis

A two-stage clinical trial design was used to determine sample size, resulting in two staged cohorts of dogs being enrolled in the study. Data were collected on 10 dogs randomly distributed between the two treatment groups. A sample size of 14 dogs/group was calculated based on the symmetry indices of TPI at 2 weeks (0.63 versus 0.48; SD = 0.14), a power of 80% and a probability of type I error of 0.05.

Continuous data (cost and duration of surgery, range of motion, girth circumference, maximum temperature, and symmetry indices) were tested for normality with Shapiro–Wilk tests and reported as mean (standard deviation). Nonparametric data (owner score, lameness score, cranial drawer, and OA score) were reported as median (range). Normally distributed, continuous data were compared between treatments at each time point with Student’s *t*-test and the Wilcoxon signed rank test for nonparametric data or non-normal distributions. Changes in owner’s scores, girth circumference, and radiographic scores across time were expressed as a percentage of the ratio (postoperative value ─ corresponding preoperative value)/preoperative value and compared between groups. The significance level was set at 0.05.

## Results

### Demographics

A total of 36 dogs were screened to identify 24 meeting the criteria for inclusion in the study. Other dogs were excluded due to concurrent patellar luxation (7 dogs), neurological deficits (one dog), femoral angular deformity (one dog), inability to walk due to bilateral CCLD (one dog), and body weight over 60 Kg (2 dogs). The study population consisted of 12 male neutered, one male, and 11 spayed female dogs. Their mean age and weight were 5.2 ± 2.7 years and 24.6 ± 13 Kg, respectively. No demographic difference was detected between treatment groups. Breeds included 14 mixed breeds, two Australian Shepherds, two Labrador Retrievers, one Akita, one Australian Cattle dog, one German Shepherd, one American Pitbull, one Labdoodle, and one Poodle.

### Preoperative and intraoperative findings

No difference was detected between the owner’s score (median: 57%, min-max: 6–93%), lameness scores (3, 1–4.5), cranial drawer (7 mm, 5–15), stifle flexion (39.13 ± 8.28°), and extension (145.70 ± 11.18°) between groups. The only preoperative difference consisted of a greater OA score (12/96, 2.5–31) in dogs treated with ECR compared to those treated with pLFS (4/96, 2.5–17.5; *p* = 0.01). Medial meniscus tears (four radial and one bucket handle) were diagnosed during exploratory arthrotomy in five dogs, all treated with a partial meniscectomy and ECR. The pLFS technique was faster (62.3 ± 14.8 min) than the ECR repair (80.8 ± 14.5 min, p = 0.01) by approximately 20 min. The cost of pLFS ($557.7 ± 89.6) was lower than that of the ECR ($646 ± 95.3, *p* = 0.03) by approximately $100.

### Postoperative outcomes

All dogs completed the 12-week-long study. The only major complication encountered consisted of postoperative septic arthritis diagnosed at 2 weeks after pLFS. The dog had become non-weight-bearing, the stifle was swollen, and a purulent discharge was present over the surgical incision. *Staphylococcus pseudointermedius* and *Staphylococcus aureus* were isolated from the joint. The extracapsular repair was removed, and the joint was lavaged. A tibial plateau leveling osteotomy was performed after a course of antimicrobial therapy and negative bacterial cultures. The dog regained its limb function, but the statistical analysis included only the measurements obtained before and immediately after surgery. Another dog treated with pLFS was diagnosed with a surgical site infection 2 weeks after surgery and was successfully treated with antimicrobial therapy. All data were collected on this dog and included in the analyses.

**Figure 3:**
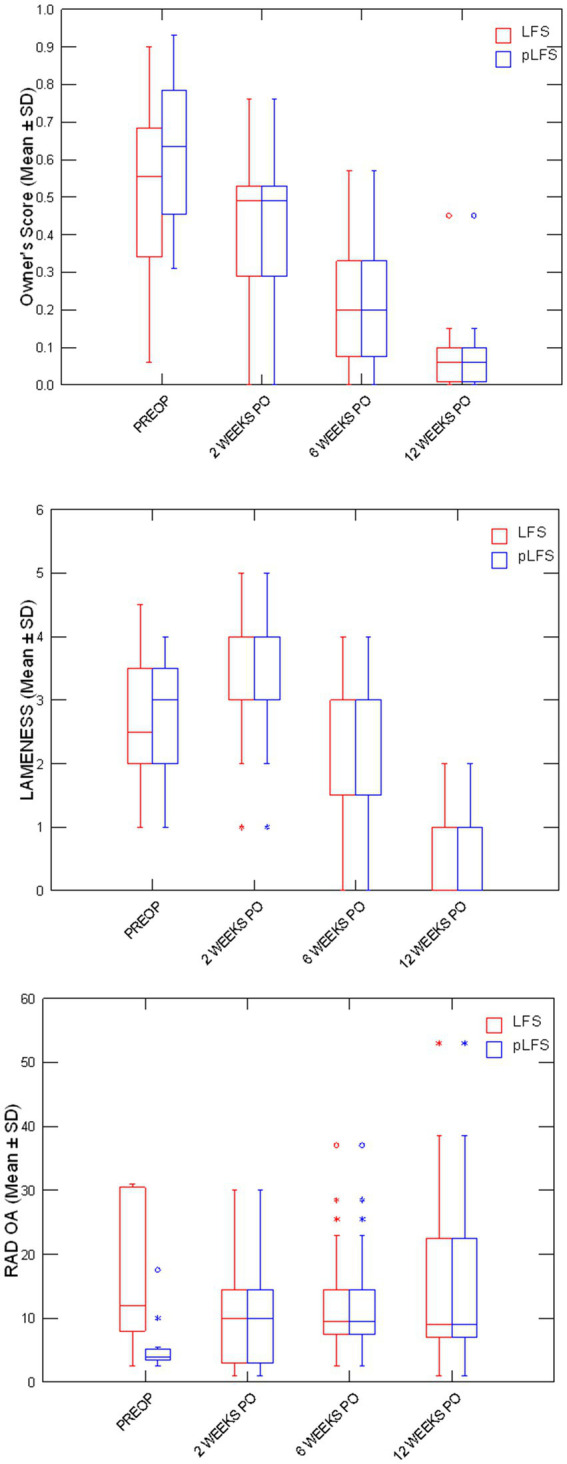
Boxplots representing medians (50%) and 75% quartiles for owners’ scores, lameness scores, and osteoarthritis scores (OA) between treatment groups at each time point. * *p* < 0.05 between groups.

**Figure 4:**
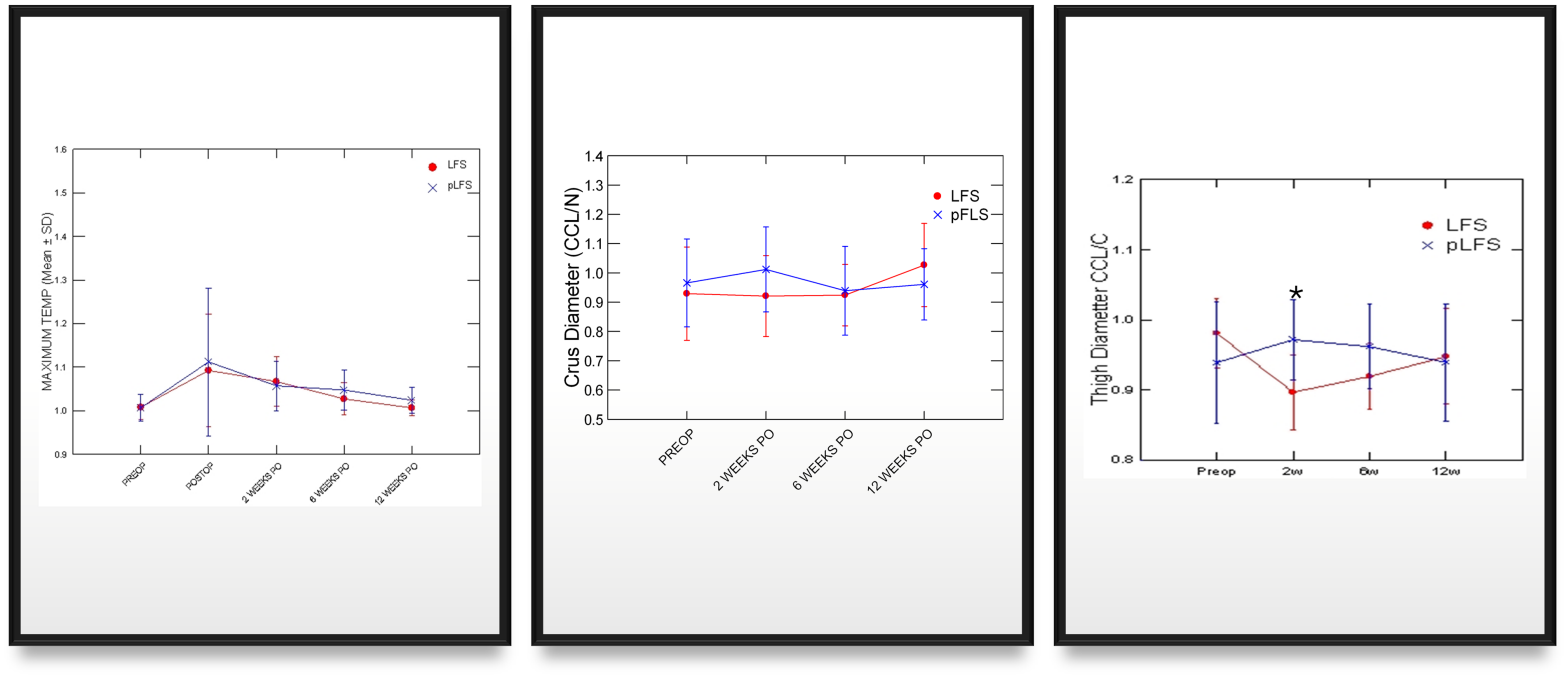
Graphic representations of means (SD) girth and thermal imaging measurements across time after traditional (ECR) or percutaneous (pLFS) lateral fabellar suture repair of CCLD. Maximum temperature: Ratio of the maximum temperature over the affected stifle to that of the contralateral stifle measured via thermal imaging. Thigh Diameter: Ratio of the thigh diameter of the affected limb to that of the contralateral limb. * *p* < 0.05 between groups.

Owners assessments improved over time (Figure 3) without difference between groups, decreasing from 57% (21–93%) preoperatively to 6% (0–15%) at 12 weeks after surgery. The cranial drawer was eliminated in all dogs immediately after surgery and remained between 0 and 2 mm throughout the study. Lameness scores initially increased after surgery (*p* < 0.0001) as all dogs except one, treated with pLFS, were non-weight-bearing (lameness score of 5/5) on the operated limb the day after surgery. No statistical difference was detected between lameness scores of dogs treated with ECR or pLFS at any time point (*p* = 0.3), although the median lameness scores were 3/5 (1–4) 2 weeks after pLFS and 4/5 (2–5) after ECR. Flexion was improved at 2 weeks in stifles treated with pLFS (36.1 ± 6.3°) compared to ECR (42.8 ± 10.7°, *p* = 0.04). In contrast, extension of stifles operated with ECR (143.4 ± 7.9° and 146.1 ± 8.4°) exceeded that of limbs treated with pLFS (134 ± 9.3° and 136.5 ± 8.2°, *p* < 0.02) by approximately 10° at 6 and 12 weeks after surgery. The only difference in muscle mass detected throughout the study (Figure 4) consisted of a smaller reduction in thigh diameter (−1.2 ± 0.05% compared to preoperative values) 2 weeks after pLFS compared to ECR (−5.5 ± 0.04%, *p* = 0.03).

**Figure 5:**
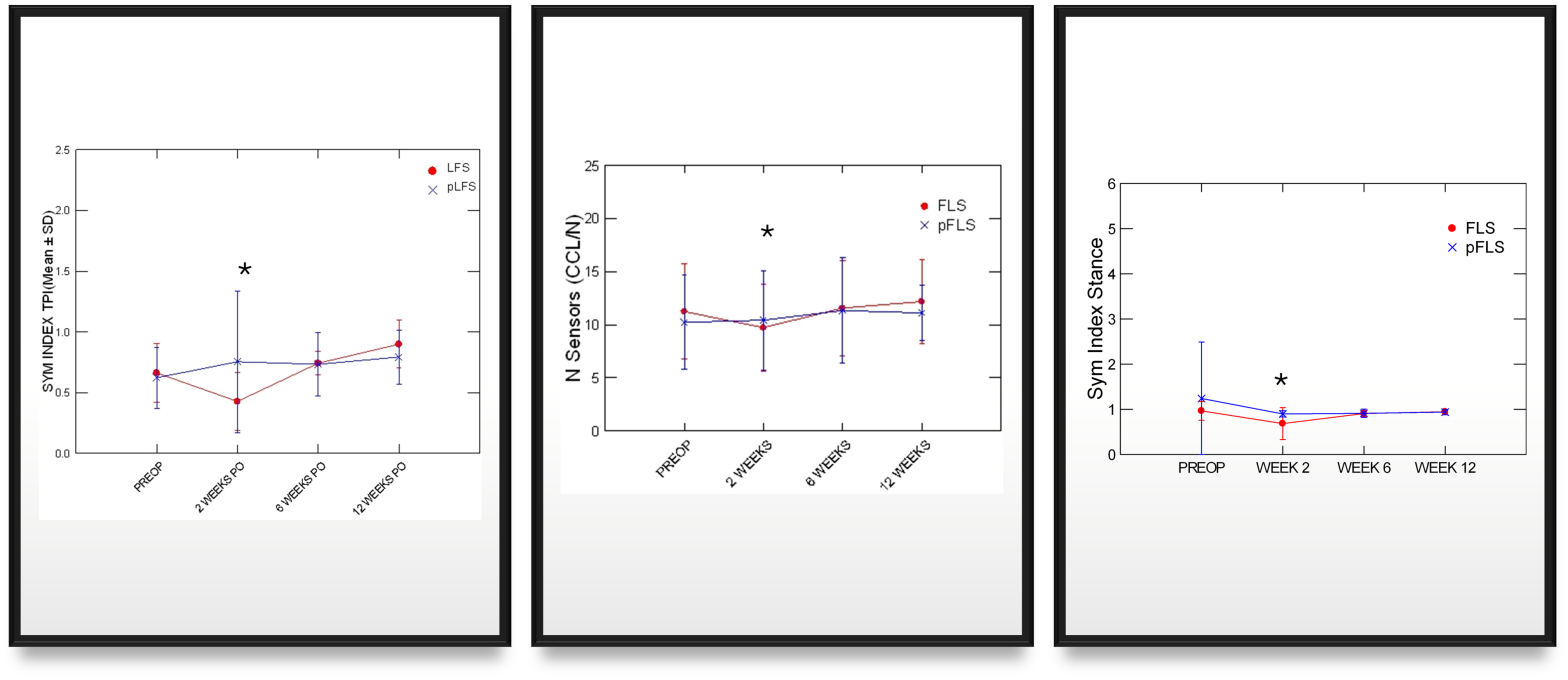
Graphic representations of means (SD) of symmetry indices generated via pressure gait analysis across time after traditional (ECR) or percutaneous (pLFS) lateral fabellar suture repair of CCLD. SYM index: Symmetry of the total pressure index between the operated and contralateral limb, measured via pressure gait analysis. Nb of Sensors: Number of sensors activated by the operated limb. Stance Ratio: the % of the stride spent in stance by the affected is divided by that of the contralateral limb. * *p* < 0.05 between groups.

Adequate placements of the tibial tunnel and crimp clamps were confirmed on postoperative radiographs and remained unchanged throughout the study. The OA scores remained greater in dogs with ECR than pLFS (*p* < 0.01, Figure 3) at each time point. However, no difference was detected in the progression of scores normalized to their preoperative values between groups at 12 weeks (*p* > 0.1). The maximum temperature recorded over the operated stifle increased 24 h and 2 weeks after surgery in both groups compared to preoperative values. However, no difference was detected between treatment groups throughout the study (Figure 4). Symmetry indices were greater at 2 weeks after pLFS than ECR (Figure 5). The pressure placed on the operated limb averaged 78% of that of the contralateral limb after pLFS compared to 43% after ECR (*p* = 0.04). The ratio of sensors activated by the operated limb was approximately 25% greater after pLFS than ECR (0.76 ± 0.24 and 0.52 ± 0.26, respectively, *p* = 0.02). Similar results were obtained when comparing the relative stance time of operated limbs (0.90 ± 0.06 s after pLFS; 0.68 ± 0.34 s after ECR, *p* = 0.03). No other difference was detected between treatment groups at the walk throughout the study and the trot at 12 weeks. At that time, the TPI indices were 0.71 ± 0.31 in the pLFS group and 0.75 ± 0.15 in the ECR group (*p* = 0.7), the ratios of activated sensors between treated and contralateral limbs were 0.83 ± 0.21 after pLFS and 0.85 ± 0.11 after ECR (*p* = 0.3), and the ratios of stance time were 0.92 ± 0.1 after pLFS and 0.94 ± 0.05 after ECR (*p* = 0.6). All indices were at least equal to 1 in two dogs treated with pLFS and one dog treated with ECR.

## Discussion

The main findings of this study are: (1) placing a pLFS in CCLD stifles was faster and more cost effective than the traditional extracapsular repair; (2) Girth circumference and pressure gait analyses at 2 weeks provide evidence to suggest an earlier return to function in limbs treated with the pLFS; (3) No difference was detected in thermal imaging between treatment groups. Combined, these results justify the acceptance of our hypotheses that the pLFS was faster and resulted in an earlier return to function compared to traditional LFS. However, the thermal imaging results do not support the hypothesis related to reduced postoperative inflammation.

The study was designed to treat dogs in low-cost settings, and dogs belonged to owners who had declined alternative treatment options due to financial constraints. An injectable anesthesia protocol was used dogs undergoing pLFS, supplemented by Sevoflurane, as needed to maintain an adequate plane of anesthesia. This agent was selected based on its availability in the clinic and faster recovery. However, it could be replaced in most patients by Isoflurane to reduce the cost of anesthesia. The implants (crimp clamps and suture material) and technical assistance fee were similar between procedures. The cost of pLFS, including implants, was about 100$ lower than that of the ECR, when the surgical professional fee was solely based on duration. The gap between treatment costs would widen if technical assistance and anesthesia were also charged per duration or if an additional fee were added for the exploratory arthrotomy. Crimp clamps were selected to secure NLL ≥ 18Kg because they have been found to increase load to failure and stiffness, while reducing elongation of LFS compared to knotted constructs (21). This effect was not detected when small NLL was placed, justifying manual knots. Similarly, an individually packaged double-swaged-on nylon suture was purchased to control the biomechanical properties of the extracapsular support. Surgical outcomes may differ with suture material and sterilization method (22–24).

Dogs were included in the study if they were diagnosed with unilateral lameness associated with cranial drawer and joint effusion. These signs, combined with the absence of clinical signs of other stifle conditions (such as patella luxation) and favorable postoperative outcomes, support the diagnosis of a deficient CCL in limbs treated with pLFS. The postoperative function was improved 2 weeks after pLFS compared to ECR, as documented by girth diameter and pressure gait analysis. Thermal imaging did not allow the detection of differences in surgical site inflammation between groups. This technique lacks specificity as measurements are influenced by numerous factors, including room and dog temperature, clipping, distance, and hair length. These variables were standardized in our study, and measurements were normalized to those obtained on the contralateral limbs. However, hair length over the surgical site varied throughout the study, which could influence longitudinal comparisons within dogs and contribute to the increased temperatures recorded immediately and 2 weeks after surgery. Variation in hair regrowth between dogs could have contributed to a type II error in our thermal imaging results. Function was likely improved by the reduced disruption of soft tissues and associated pain in limbs treated with pLFS. The respective influence of the absence of exploratory arthrotomy versus the percutaneous placement of the suture in the pLFS group cannot be determined since both were combined. Extending the soft tissue incision between the two incisions of the pLFS while preserving the joint capsule would facilitate the suture placement and allow imbrication of the Fascia Lata. This imbrication has been proposed to improve joint stabilization, but a biomechanical study found no influence of periarticular tissues on the craniocaudal stability of normal stifles and CCLD joints stabilized with an intracapsular repair (25). However, this technique is likely to cause a painful postoperative fasciitis since the initial phase of tissue healing consists of inflammation, associated with pain.

Stabilization of the CCLD joint without arthrotomy has previously been reported in 696 TPLOs with good long-term clinical outcomes and fewer complications than joints explored through a medial arthrotomy (26). Other proposed advantages included reduced postoperative pain, faster surgery, and reduced cost. Meniscal tears in humans can be asymptomatic and have been reported in 56% of MRI studies of men over 70 (27). The management of meniscal lesions remains controversial, with a reverse trend from arthroscopic to conservative management since 2011 (28, 29). Two systematic reviews reported no difference in long-term outcomes when degenerative non-obstructive meniscal tears were treated conservatively compared to arthroscopic surgery (28, 29). In dogs, partial meniscectomy has been found to accelerate the progression of degenerative changes in the stifle (30), but the conservative management of meniscal lesions remains poorly documented. The inability to assess meniscal disease in joints treated with pLFS in our study could increase the risk of postoperative lameness. However, our findings do not provide evidence of such complication as function did not differ between groups at the walk or the trot 12 weeks after surgery.

Several limitations justify a cautious interpretation of our results. First, the absence of exploratory arthrotomy precluded the assessment of menisci and the excision of the CCL remnants in dogs treated with pLFS. This limitation is inherent to the technique but prevents us from eliminating meniscal disease as a confounding variable in the study. Nanoscopy or arthroscopy can be combined with a pLFS to preserve the advantages of a minimally invasive repair while assessing and treating meniscal disease and trimming the remnant of the CCL. This approach would reduce the risk of recurrent lameness due to postoperative meniscal disease and would be appropriate for clients who do not opt for a tibial osteotomy but could afford this treatment. However, our study did not use these techniques because equipment cost and technical complexity preclude their application in a low-cost setting. Meniscal lesions were identified in 5/12 dogs treated with ECR; these could account for this group’s higher preoperative OA scores. However, radiographic evidence of degenerative joint disease does not correlate well with function (31), and no preoperative difference in function was identified between groups based on girth, goniometric, and pressure gait measurements. The absence of detectable differences in preoperative function and postoperative progression of OA between groups allows comparison of postoperative function between groups. This comparison is limited to 12 weeks, precluding long-term comparison between ECR and pLFS. The main difference in long-term outcomes would likely stem from a higher risk of post-liminary meniscal disease after pLFS than ECR, although gross evaluation of the stifle has not been found as sensitive as arthroscopy to detect meniscal disease (32). The duration of follow-up also precludes conclusions regarding the loosening of the LFS. However, the same risk of a recurrent cranial drawer can be expected since the placement of the lateral fabellotibial suture was similar between the two techniques.

The outcomes of dogs treated with extracapsular repairs are well documented in the literature and include objective assessments of postoperative function. No difference in clinical outcomes was reported in a prospective observational study of 65 medium to large dogs (weighing 10–60 Kg) with CCLD assessed with ground reaction forces at the walk and radiographic progression of OA over 24 months after TPLO or ECR (14). Another study reported similar results in Labrador Retrievers evaluated at the walk 6 months after ECR (47 dogs) or TPLO (64 dogs, (33)). However, recent publications conclude that CCLD has superior functional outcomes when treated with tibial plateau leveling osteotomies rather than traditional ECRs (4, 10, 34). Differences seem more noticeable in larger dogs, stifles with excessive tibial plateau angles (>30°), and when the gait is evaluated at the trot (10, 34, 35). By contrast, most clinical reports of outcomes of dogs treated with ECR describe high owner satisfaction rates (82 to 90%) and apparent resolution of lameness (in 78–82% of dogs) (36–38). A 3D-kinematic study of nine medium to large dogs treated for complete CCLD described the persistent cranial translation of the tibia 6 months after ECR with a knotted nylon leader material (39). However, owners’ assessments and the clinical lameness scores of these dogs remained improved. It is clear from this evidence that kinetics and kinematics are more sensitive than subjective clinical evaluations and allow the detection of subclinical lameness. This evidence also suggests that ECR can lead to acceptable levels of function in selected candidates. This study does not intend to propose pLFS as an alternative to tibial osteotomies or as a gold standard for treating CCLD in dogs of any size. Instead, the results reported here provide evidence to support the placement of a pLFS as a cost effective alternative to accelerate return to function in candidates to conservative treatment or conventional ECR of CCLD.

## Data Availability

Datasets are available on request: The raw data supporting the conclusions of this article will be made available by the authors, without undue reservation.
